# Supplementary material for: The interactome of intact mitochondria by cross-linking mass spectrometry provides evidence for coexisting respiratory supercomplexes
Source: Mol Cell Proteomics. 2017 Dec 8;17(2):216–32. doi: 10.1074/mcp.RA117.000470 (PMC5795388; doi:10.1074/mcp.RA117.000470)
Supplement: Supplemental Data [file supp_RA117.000470_133922_0_supp_23978_fzffwf.zip › spectra_annotation/mito_spectra_annotation/292-1-7-1-32-1.pdf]

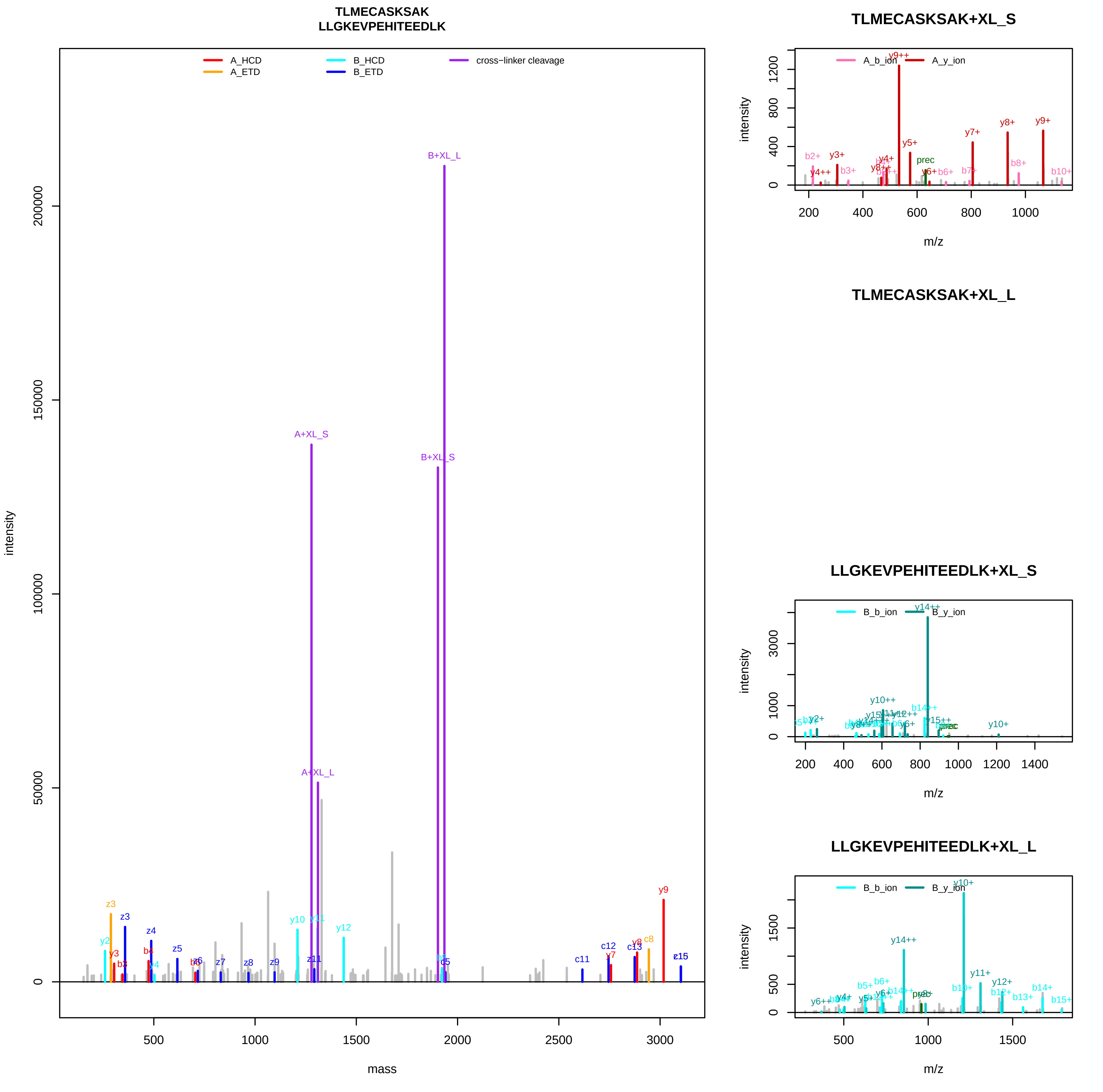
TLMECASKSAK
LLGKEVPEHITEEDLK
A_HCD
A_ETD
B_HCD
B_ETD
cross−linker cleavage
B+XL_L
A+XL_S
B+XL_S
A+XL_L
y9
c15
c12
c13
c11
c8
y7
y10
y11
y12
z11
z3
z4
z5
z6
z7
z8
z9
y2
y3
b3
b4
b6
y4
c5
intensity
mass
0
50000
100000
150000
200000
500
1000
1500
2000
2500
3000
TLMECASKSAK+XL_S
A_b_ion
A_y_ion
y9++
y9+
y8+
y7+
y5+
y3+
y4++
b2+
b3+
b7+
b8+
b10+
prec
y6+
b6+
m/z
0
400
800
1200
200
400
600
800
1000
TLMECASKSAK+XL_L
LLGKEVPEHITEEDLK+XL_S
B_b_ion
B_y_ion
y14++
y10++
b14++
y15++
y10+
y2+
y6+
prec
0
1000
2000
3000
200
400
600
800
1000
1200
1400
LLGKEVPEHITEEDLK+XL_L
y10+
y14++
y11+
y12+
b10+
b12+
b13+
b14+
b15+
b5+
b6+
y4+
y6++
y5+
prec
0
500
1000
1500
500
1000
1500
